# Behaviour of Rectangular Hollow Thin Ply Carbon Thermoset and Thermoplastic Composite Tubes Subjected to Bending

**DOI:** 10.3390/polym14071386

**Published:** 2022-03-29

**Authors:** Somen K. Bhudolia, Goram Gohel, Durga Vasudevan, Kah Fai Leong, Pierre Gerard

**Affiliations:** 1 School of Mechanical and Aerospace Engineering, Nanyang Technological University, 50, Nanyang Avenue, Singapore 639798, Singapore; goram001@e.ntu.edu.sg (G.G.); mkfleong@ntu.edu.sg (K.F.L.); 2 Technical University of Munich Asia, 25 International Business Park Rd, Singapore 609916, Singapore; dv.527699@tum-asia.edu.sg; 3 Groupement de Recherche de Lacq, Arkema, Route Départementale 817, BP 34, 64170 Lacq, France; pierre.gerard@arkema.com

**Keywords:** hollow composite tubes, thermoplastic resin, bending, failure mechanisms

## Abstract

Tubular composites are widely used in many industrial applications, and there is need to use new material and reliable manufacturing processes to improve the performance and process aspects. The current research presents a detailed study to understand the flexure response of rectangular tubular composites based on thin ply carbon fibres and Elium^®^ resin. Another aim was to understand the failure mechanisms of novel tubular thermoplastic composite systems and carry out a baseline comparison with Epoxy-based tubular systems. In the current research, a bladder-assisted resin transfer moulding process was used to manufacture hollow thermoplastic composite tubes, and the bending behaviour of thin ply carbon (TPC) composite parts with novel Elium^®^ (EL) and Epoxy (EP) resin as the matrix material was studied using a detailed experimental study. A testing method with optimized support span and a saddle was used to carry out three-point bending tests on the tubular composite structures. The TPC/EL composite tubes have shown 10% higher bending strength, with a noticeable increase in deformation due the presence of extended plasticity attributes for acrylic Elium resin. Failure mechanisms studied with the detailed microscopic investigation have shown severe catastrophic failure for epoxy-based composite tubes; however, acrylic Elium^®^-based composite tubes have shown different damage modes such as fibre splitting, resin infragmentation, and fibre resin-interfacial cracking.

## 1. Introduction

Hollow composite structures are extensively used in many engineering applications such as marine, automobile, aerospace industries and especially in sporting equipment [1,2]. From an engineering point of view, fibre provides outstanding properties in an in-plane direction (*x*, *y*-axis). Additionally, the resin system plays a crucial role in the thickness direction (in the *z*-axis direction) and provides support to hold the fibres to reduce delamination between the layers. The type of resin also determines the manufacturing processes, pressure, and temperature limits. The appropriate usage of both is very significant to achieve a composite system with optimized mechanical properties. Hollow composite structures can be manufactured by different processes. The most widely used processes are filament winding process, pultrusion process, and bladder resin transfer moulding process [3]. Inflatable bladder-assisted resin transfer moulding (B-RTM) was chosen for better surface finishing quality and uniform thickness [4].

Thin carbon fibre plies are available in thicknesses from 0.12 mm to 0.5 mm, and as per the European patent, thin plies are the ones with a thickness of one-sixth of the thicker plies. For the thin plies spreading, the first ply is oriented in one specific direction, and the next layer is placed with respect to the first ply direction. Composites that are manufactured from thin plies possess excellent capabilities in both static and dynamic load cases to overcome microcracking and delamination challenges [5,6,7,8]. In carbon-based composites, the structures fail at 2% strain, but micro-cracks initiate at an early strain level of 0.5% [5]. Thermoplastic resins are challenging to use due to the higher associated temperature curing cycles [9,10]. However, they offer improved damping [11,12], higher crack critical energy rates to arrest crack propagations [13,14], reshapability [15], impact and damage tolerance [16,17,18,19,20,21], the ability to be welded [22,23,24,25], and are recyclable [26,27]. Elium^®^ liquid resin is a recent invention from ARKEMA to manufacture thermoplastic composite parts. It is available in a liquid state with low viscosity and possesses significantly higher impact, fracture toughness, and vibration damping attributes [12,18,22,23,24,25,28,29,30,31,32,33,34,35].

Dejun Yin et al. studied the flexural response of carbon fibre-reinforced tubes filled with Al foam and showed that the bending stiffness and the energy-absorbing capability of the tubes with the addition of foam fillers increased, and the increase was more substantial with the increase in the foam density of the metal [36]. In another study, the performance of flax-reinforced epoxy composite tubes is studied by adopting the four-point bending test [37]. Libo Yan et al. demonstrated from their study that the results at the tubular and the flat scale are very similar to brittle catastrophic failure. It was also concluded from their study that the increase in thickness leads to a simultaneous increase in the load and deflection to failure characteristics. Ali Amiri et al. carried out research investigating the benefits of hybridization of the carbon fibres with the flax fibres [38]. They showed that there was an increase in the load-carrying ability with hybridization, but it cannot be fully determined whether the added benefits are due to the addition of flax fibres or the higher thickness in the case of the hybridized tube. M. Stefanovska et al. investigated the flexural performance of glass/epoxy tubular composites manufactured using the filament winding process [39]. The effect of winding angle was studied, and the bending stiffness was found to be increased with the decrease in the winding angle. The failure mechanisms involved matrix cracking, fibre splitting, and inter- and intra-ply delamination. Zhenyu Wu et al. have investigated the gradient effect on the bending response of over-braided composite tube with two layers of ±40° layers [40]. The layer that was in direct contact with the loading pin had a detrimental effect on the flexural properties and the damage mechanisms. Bhudolia et al. investigated the bending response of carbon acrylic Elium^®^ composite tubes and compared its performance with epoxy-based composite parts [12,41,42]. The flexural properties were very similar, barring the ability of the acrylic-based composite to take larger displacement before the failure, but the details of the failure mechanisms were not studied. The current research presents a detailed study to understand the flexure response of rectangular tubular composites based on thin ply carbon fibres and Elium^®^ resin. It was also intriguing to develop a detailed interpretation of the failure mechanisms of novel tubular thermoplastic composite systems and carry out a baseline comparison with Epoxy-based tubular systems; hence, a detailed microscopic investigation is also carried out.

## 2. Materials and Manufacturing

**Figure 1:**
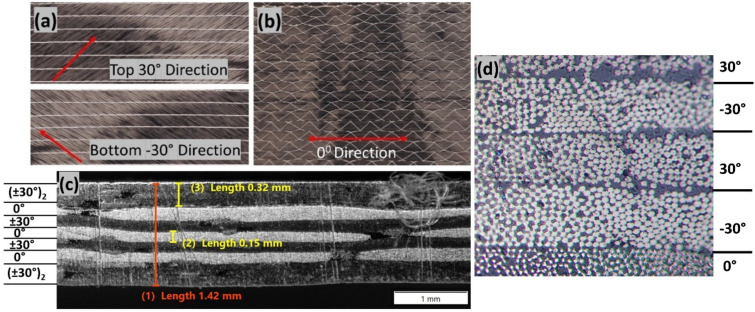
(**a**) BX 30 C−ply^TM^ fabric (**b**) UD (0°) fabric (**c**,**d**) microscopic images showing the individual layers and the cross-sectional details.

Composite tubes are manufactured using a combination of uni-directional thin ply (144 gsm) and C-Ply^TM^ BX30 bi-angle ply (150 gsm) (Refer Figure 1a,b) [41]. Thermoplastic Elium^®^ 150 acquired from Arkema, France was used as a matrix system. Elium^®^ resin has a viscosity of 100 cP and is in a liquid state at room temperature (RT) [18,43,44,45]. On the other hand, similar to thermoplastic composites, the same fibre architecture but with epoxy sizing was used to manufacture the thermoset composite tubes. Epoxy (AM-8937 A/B) resin used as a thermoset matrix was obtained from Wells Advanced Materials Co. Ltd, Shanghai, China. Figure 1c,d show the individual layer thicknesses and the angles under the microscope.

**Figure 2:**
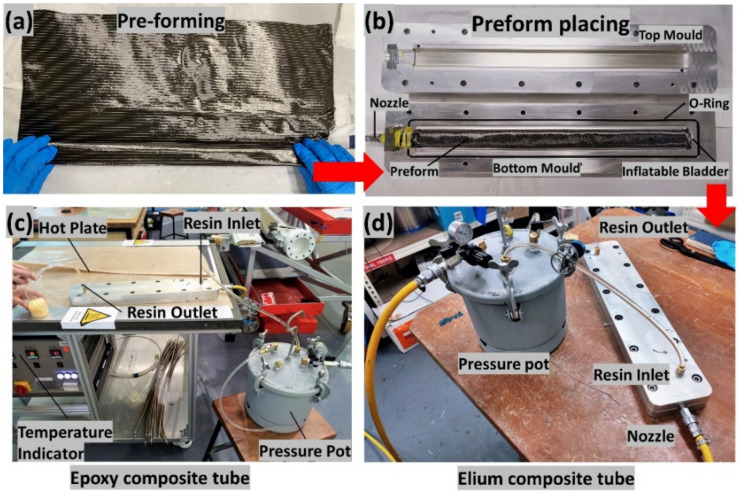
B-RTM manufacturing steps and set-up.

Thermoset and thermoplastic composite tubes were manufactured using the bladder resin transfer moulding (BRTM) manufacturing process. Several samples were manufactured to optimize the injection and bladder pressure with thin ply fabric tubes impregnated with either thermoplastic or thermoset resin. The overall process steps of B-RTM are shown in Figure 2.

A two-part aluminium mould was used for the manufacturing of tubular composites. For manufacturing of the tubular composite, the first step was mould preparation; the mould release was applied onto the mould surface for ease of the demoulding of the manufactured part, followed by dry fibre preforming. Preforming of dry fibres was carried out over the mandrel to obtain the fibre layup of [(±30°)_2_/0°/±30°/0°/±30°/0°/(±30°)_2_] (refer Figure 2a). Once the preform was made, it was taken out of the mandrel and was placed into the mould, as shown in Figure 2b. The mould was then closed, and the bladder was initially inflated (3 bar) to properly compact the fibres over the mould. Then, the resin was injected (2.8 bar) into the mould at the required injection pressure. Once the part was filled, the injection was stopped, and the bladder pressure was further increased for consolidation (5 bar) to allow the excess resin to squeeze out and to obtain the uniform thickness and Vf (54%) of the part. To manufacture the thermoplastic composite part, injection was carried out at room temperature (RT), followed by post-curing at 65 °C for 1 hr (refer Figure 2c). For epoxy composite, the injection was carried out with mould at 50 °C (refer Figure 2d). Once the part was filled, the mould temperature was raised to 100 °C for curing for 10 min, followed by cooling to RT and demoulding. The final thickness (t) and the fibre volume fraction (V_f_) of the manufactured composites tubes were 1.4 ± 0.2 mm and 54 ± 0.93%, respectively. Additionally, ASTM D792 [46] and ASTM D2734-09 [47] tests were carried out, and the porosity for the manufactured tubes was found to be less than 1% and was used for mechanical testing. The two different configurations investigated are Thin ply_Carbon/Elium^®^ (TPC/EL) composite and Thin ply_Carbon/Epoxy (TPC/EP) composite.

## 3. Experimental Setup

**Figure 3:**
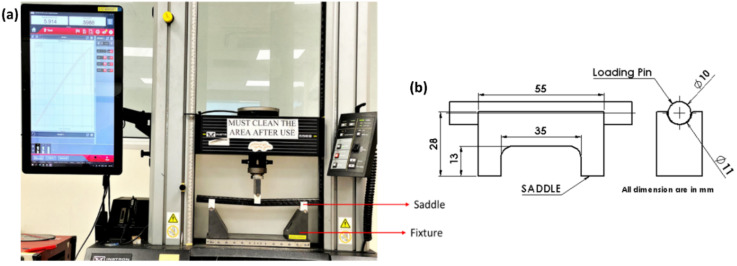
(**a**) Flexure experimental test set-up with saddle supports for testing tubular composites (**b**) dimensions of the saddle and the loading pin.

The flexural properties of the composite rectangular tube were investigated following the ASTM D 790 [8] standard. The three-point bending flexural testing of the composite tube using the saddle support was performed using an Instron 5569 machine (refer Figure 3a). As shown in Figure 3a, the saddles were positioned onto the rollers of the fixture, and tubes were placed into the saddles and are aligned. The dimensions of the saddle and the loading pin are shown in Figure 3b. The load (kN) and displacement (mm) data are acquired from the Bluehill 4 software from the machine. The bending response of the tubular composite sample is highly dependent on the support span of the specimen during the test. Currently, the widely used ASTM D790 standard is suitable for flat laminate testing and may not be directly applicable to tubular geometries. However, many researchers have used a similar standard to determine the fundamental flexural response of the tubular structures [39,40,48]. Support span is essentially required to be optimized for the geometry under consideration to avoid shear and localized bending and crushing effects. These effects dominate when a shorter support span is used during the testing and must be eradicated. Damage analysis was carried out on the failed samples under flexure using an optical microscope (OLYMPUS SZX7) to understand the failure mechanism.

## 4. Results and Discussion

For flexural testing, two different configurations were investigated: TPC/EL and TPC/EP composite tube. During flexure testing of the tubular structures, the cracks were propagated in both transverse and longitudinal directions. Flexure tests were carried out to calculate the bending stresses and the modulus of the manufactured tubular configurations. It was also important to understand the differences in the flexural behaviour along with the failure mechanisms of the composite tubes manufactured with Elium^®^ and Epoxy resin systems. One of the key factors that has a major effect on the flexural behaviour of the composite structures is the support span used during the testing. Following the standard [49], the maximum recommended span to thickness ratio of 60:1 was chosen in the current investigation. A larger support span was used because it eliminates the possible shear effect, considering the peculiar nature of the geometry under consideration as well the concentrated and undesired localized bending.

**Figure 4:**
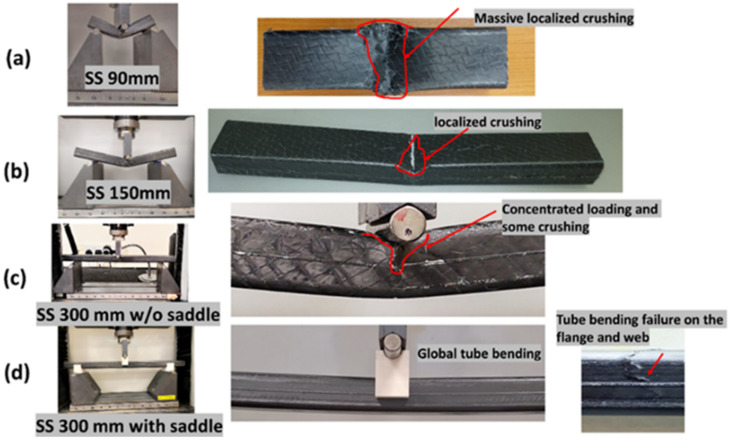
(**a**–**d**) Tube bending failure behaviour with different support span and the use of saddle.

**Figure 5:**
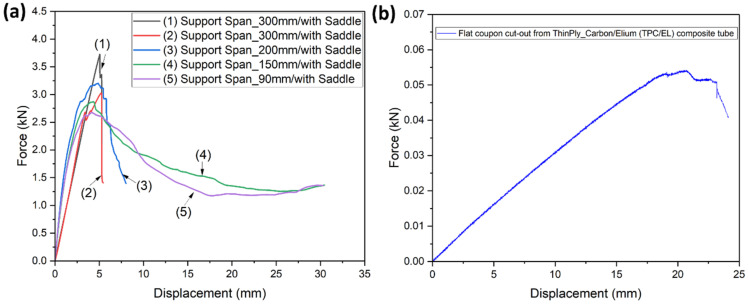
Load vs. displacement curve for (**a**) TPC/EL composite tube with different support spans and with the saddle arrangement (**b**) flat coupon cut-out from TPC/EL composite tube.

The maximum thickness of the manufactured tube under consideration was 1.4 mm, and hence the first tests were carried out with a support span of 90 mm with an overhanging length of 30 mm on each side of the support rollers. This overhanging length was always ensured to be at least 10% of the support span following the ASTM D790 [49]. The initial study was carried out using one TPC/EL configuration, and once the optimised support span was defined, the comparison study was carried out for different tubular configurations using the optimised support span. Two strategies were considered to optimise the real bending scenario of the composite tube: (1) by increasing the support span (refer Figure 4a,c) and (2) using saddles. Saddles, as shown in Figure 4d, were used to reduce the concentrated effect of the roller pins on the composite tube created during the bending rather than creating a global bending behaviour. Support spans of 90 mm, 150 mm, 200 mm, and 300 mm were used in the current investigation, with a minimum overhanging length of at least 10% of the corresponding configuration. The load–displacement curves for the different configurations of TPC/EL composite tubes are shown in Figure 5a. The tubes tested with a support span of 90 mm and 150 mm without using a saddle have shown a peak load of 2.62 kN and 2.85 kN, respectively, with a significant flexural displacement of up to 30 mm. The tube with a 200 mm support span without using a saddle has shown 3.1 kN with a flexural displacement of up to 8.5 mm. The tube tested without saddle and 300 mm support span has shown more linear behaviour until the first major failure with a peak load of 2.9 kN. Increasing the support span from 90 mm to 150 mm, it was evident from the failure modes, as shown in Figure 5a, that the samples with lower support span have undergone localized crushing rather than pure global bending of the tube. With the increase in support span, the localized crushing of the tube was seen to diminish. However, to fully eradicate the concentrated bending behaviour, the saddles were symmetrically used on both the support rollers as well as the loading pin.

The load–displacement curve for the sample with a 300 mm support span with the saddles has shown a peak load of 3.67 kN with failure mode, showing pure global bending behaviour until the complete failure of the tubular structure. Furthermore, a flat sample was cut from the tubular configuration, and flexural tests were carried out to check the flexural modulus, which is independent of the geometry of the structure (refer Figure 5b). The flexural modulus for TPC_EL_SS300_SAD configuration and the flat coupon cut from the same tube was calculated to be 36.3 GPa and 36.1 Gpa, respectively, showing good agreement between the results and the adequacy of the defined support span and the saddle arrangement for carrying out further tests on other tubular composite configurations.

**Figure 6:**
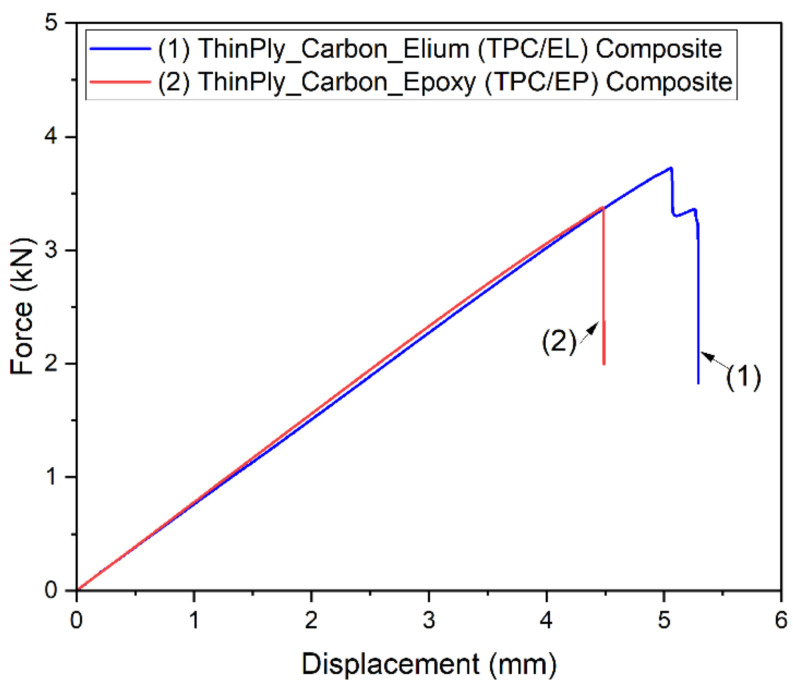
Load vs. displacement curves for TPC/EL and TPC/EP tubular composite configurations.

It can be concluded from the initial test study that the recommended span for flat specimens cannot be directly applied to tubular configurations, and there could easily be an under-estimation of the peak load and an over-estimation of the flexural modulus during the testing of the composite tubes with shorter support spans. Once the support span and the testing methodology were defined from the initial tests, a detailed investigation was carried out for TPC/EL and TPC/EP tubular composite configurations. Figure 6 shows the load–displacement curve for both the composite tubular configurations. 

**Figure 7:**
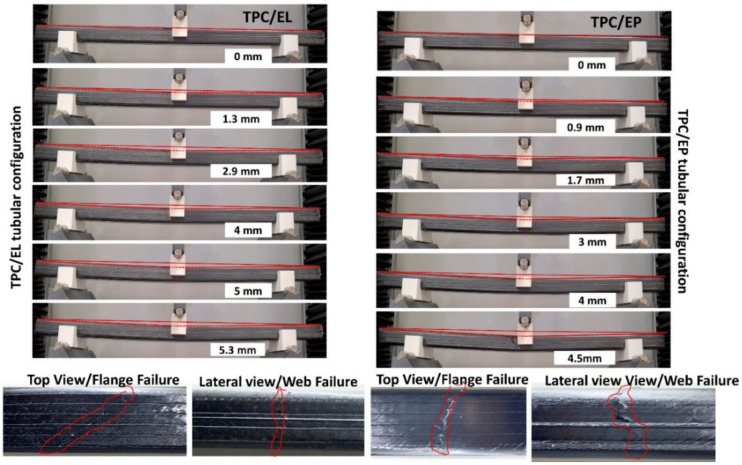
Global bending behaviour with the web and flange failure modes for the Thin Ply Carbon/Elium^®^ (TPC/EL) and Thin Ply Carbon/Epoxy (TPC/EP) composite tubes.

It should be noted that, for each tubular configuration, three tubes were tested, and Figure 6 represents the best representative curve of the three tubes, which is closer to the average values. The slope of the two configurations is very similar and hence so is the flexural modulus (refer to Table 1), whereas the TPC/EL configuration shows a 10.4% higher maximum bending strength compared to TPC/EP. It is evident from the load–displacement curve with no striations or kinks in the elastic region that there were no induced slipping mechanisms or the associated breakage of the network of molecules of the matrix. It also shows that there is synergetic deformation of the interfacial region (reinforcement/matrix), and the load is progressively transferred. The global bending behaviour of the tube can be noticed at different instances (refer Figure 7) where TPC/EL composite tube is seen to undergo pure bending and it noticeably undergoes more deformation (5.3 mm), as opposed to 4.5 mm in case of the TPC/EP configuration. The final state of failure modes for flange and web are found to be very similar for TPC/EL and TPC/EP composites, although more severe crack and localized failure in the flange was noticed for the TPC/EP composite, which must be due to the more brittle nature of thermoset epoxy matrix in the case of the latter (refer to Figure 7). The global bending at different instances of displacements and the final damage modes are shown in Figure 7.

**Figure 8:**
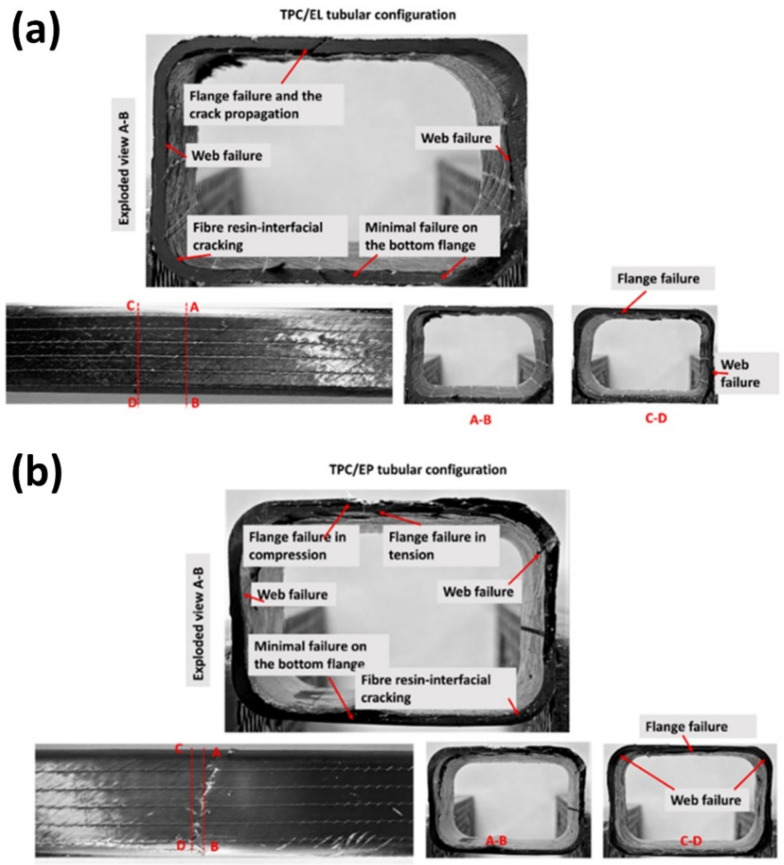
Morphology of the damaged cross-section of (**a**) TPC/EL and (**b**) TPC/EP tubular configuration at the loading position (section view) and the different cross-section along the longitudinal direction.

There is a clear improvement offered by thermoplastic Elium^®^ composite tube in terms of flexural deformation until failure when used with thin plies, as in the current investigation, which can also be related to previous studies carried out with woven reinforcement [42]. It was intriguing to further our investigation to understand the details of the failure mechanism. This was carried out by investigating the cross-section damaged morphologies of the different tubular configurations at the loading position (section view) and along the longitudinal direction (20 mm away from the loading position). Figure 8a,b show the web and the flange failures for the TPC/EL and TPC/EP configurations, respectively.

The section view of section A–B shows that the failure of the flange in the direction of the outer layer (BX30 or ±30°). There was a minimum failure on the bottom flange, whereas the load transfer from the outer flange to the web resulted in evident fibre/matrix interfacial cracks. The same feature was also seen in the cross-section C–D, with evident flange and web failure, showing that these cracks can be transferred away from the central flexural region in the longitudinal direction. Shifting the focus to TPC/EP configuration, the failure modes for the top flange are very different, with more catastrophic failure in both compression and tension. Similar features were seen for the web failure, as noticed in the case of TPC/EL configuration. For both the configurations, the downward loading of the roller caused the top surface of the flange’s failure, which is evident with the majority of ±30° and 0° layers in the TPC configuration. Herein, crack propagation is mostly in the longitudinal direction, followed by the load transfer to the web.

**Figure 9:**
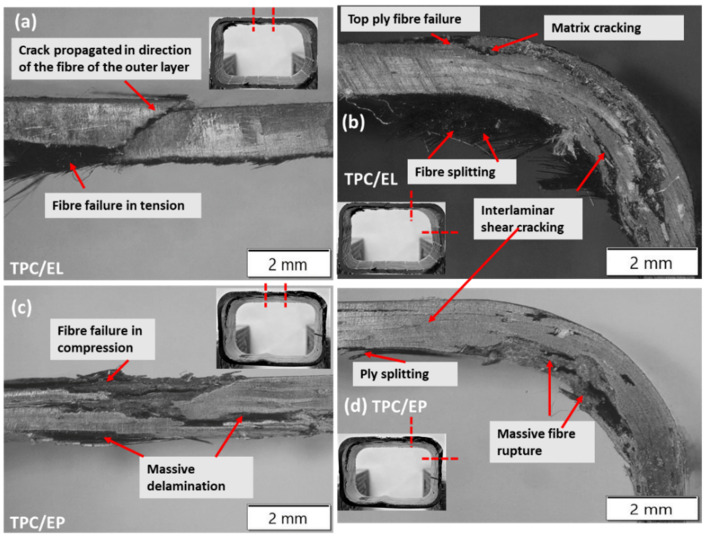
Microscopy pictures of damaged tubular C/S of TPC/EL and TPC/EP tubular configuration at the (**a**,**c**) top flange and the (**b**,**d**) top web positions.

**Figure 10:**
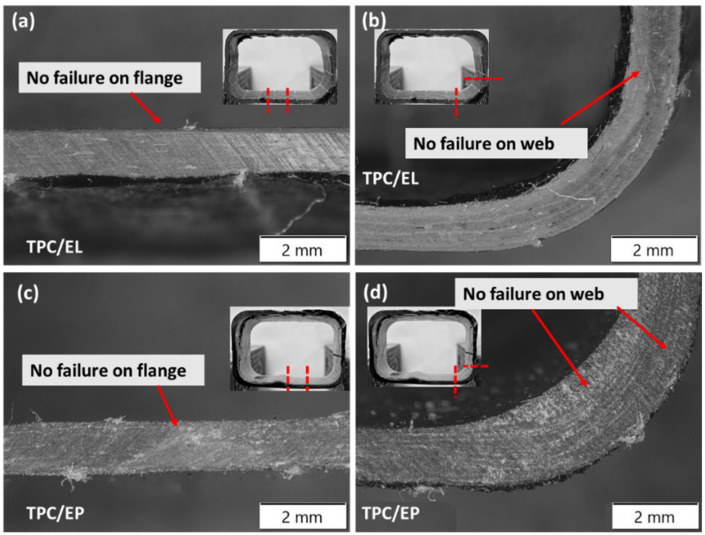
Microscopy pictures of damaged tubular C/S of TPC/EL and TPC/EP tubular configuration at the (**a**,**c**) bottom flange and the (**b**,**d**) bottom web positions.

Further, a microscopic investigation was carried out to understand the failure mechanisms in more detail. As seen from Figure 9, clear crack propagation in the direction of the fibre of the outer layer was noticed in the case of the flange of the TPC/EL composite tube, and clear tensile failure of the top flange of the tube was also noticed. The matrix cracking sites were observed on the web of the TPC/EL configuration due to the excessively higher strains developed at the corner of the tubes, which are generally more resin-rich due to the corner thickening effect in the case of the bladder moulding process (refer Figure 9) [3]. Figure 10 shows micrographs of the bottom flange and a web for the TPC/EL configuration, showing no to very minimal failure. Moving to the TPC/EP configuration, severe cracks and delamination can be seen on the top flange and the web, and the bottom flange and web also did not undergo any noticeable damage (refer Figure 10).

**Figure 11:**
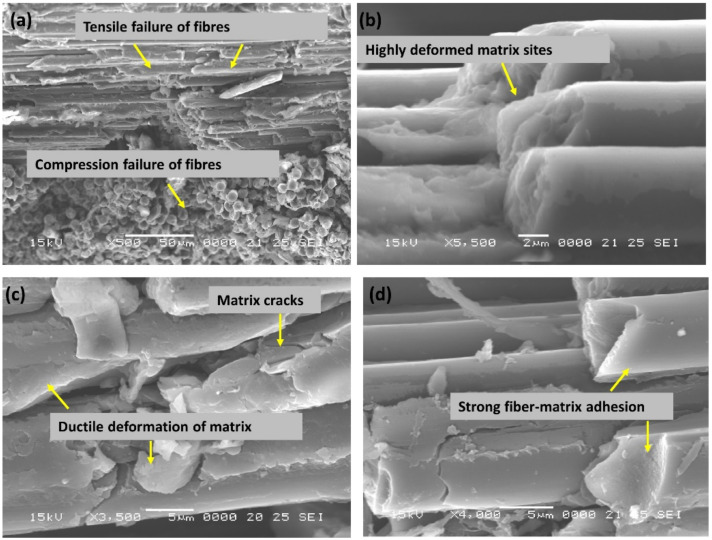
(**a**–**d**) SEM pictures of damaged TPC/EL tubular composite tube under flexural loading.

The detailed flexural study showed two important aspects that are distinguishable. Firstly, the effect of fibre architecture plays a major role in deciding the failure propagation modes. For TPC architecture, top surface failure was noticed, but there was minimal damage propagated to the bottom flange. Another important aspect is the usage of Elium^®^ resin to arrest the catastrophic failure mode [14,17,28,32,33,42,50,51,52] to a more spread failure with more flexural deformation, notably with TPC fibre reinforcement, owing to their chemical structure and presence of inherent micro ductility. SEM investion was used to confirm the distinctive thermoplastic features for Elium^®^-based composites. Whereas the flexure test is dominated by the reinforcement effect, with samples undergoing tensile and compressive failure (Figure 11a), fibre matrix interfacial bonding is enhanced in the case of thermoplastic composite due to the presence of microductility, induced due to significant plastic deformation of the acrylic Elium^®^ resin (Figure 11a–c).

## 5. Conclusions

Thin ply hollow composite tubes with Elium^®^ and epoxy resin were successfully manufactured using the B-RTM process, and their bending response was studied. It was concluded from the initial test results that the span used for flat specimens cannot be directly applied to the tubular configurations, and there could easily be an under-estimation of the peak load and over-estimation of the flexural modulus during the testing of the composite tubes with shorter support spans. Hence, an experimental setup methodology was developed by optimising the support span and the usage of saddle mechanism to induce the overall bending behaviour in the tubular composite structure. The effect of fibre architecture plays a major role in deciding the failure propagation modes. TPC/EL configuration has 10.4% higher maximum bending strength, very similar bending modulus, but has noticeably undergone more deformation owing to the extended plasticity in the case of acrylic Elium^®^ resin. The global bending behaviour of the tube was noticed at different instances where TPC/EL composite tube is seen to undergo pure bending and underwent more deformation as opposed to TPC/EP configuration. Top surface failure was noticed with minimal damage to the bottom flange, and the usage of Elium^®^ resin helps in arresting the catastrophic failure with more flexural deformation-dominated failure, notably owing to their chemical structure and presence of inherent micro ductility, which is confirmed from the SEM investigation.

## Figures and Tables

**Table 1:** Flexural test results of different tubular composite configurations under 3-point bending.

Tubular Configuration	Thickness (mm)/V_f_ (%)	Slope (from L vs. d Curve)	Maximum Bending Stress (σ_max_) (Mpa)	Flexural Modulus (E) (Gpa)	Bending Stiffness/Flexural Rigidity (EI) (Nmm^2^)
TPC/EL	1.4/54	756.61	522.42 ± 3.7	36.16 ± 1.78	425,595.2 ± 234.7
TPC/EP	1.4/54	778.90	473.31 ± 2.8	37.23 ± 1.23	438,135.8 ± 398.3

TPC/EL: Thin Ply Carbon/Elium^®^; TPC/EP: Thin Ply Carbon/Epoxy.

## Data Availability

The data presented in this study are available on request from the corresponding author.
